# Advances, Challenges, and Perspectives in Glomalin-Related Soil Protein Research

**DOI:** 10.3390/microorganisms13040740

**Published:** 2025-03-25

**Authors:** Qiumei Ling, Hanqing Wu, Lei Xie, Yuan Zhao, Qibo Huang, Qian Zhang, Ji Liu, Peilei Hu, Tiangang Tang, Jun Xiao, Hu Du, Jie Zhao, Wei Zhang, Hongsong Chen, Kelin Wang

**Affiliations:** 1 Key Laboratory of Agro-Ecological Processes in Subtropical Region, Institute of Subtropical Agriculture, Chinese Academy of Sciences, Changsha 410125, China; lingqiumei22@mails.ucas.ac.cn (Q.L.); xielei@stu.syau.edu.cn (L.X.); zhangqian@isa.ac.cn (Q.Z.); peileihu@isa.ac.cn (P.H.); tangtiangang@isa.ac.cn (T.T.); xiaojun@isa.ac.cn (J.X.); hudu@isa.ac.cn (H.D.); jzhao@isa.ac.cn (J.Z.); hbchs@isa.ac.cn (H.C.); 2 Huanjiang Agriculture Ecosystem Obervation and Research Station of Guangxi, Guangxi Key Laboratory of Karst Ecological Processes and Services, Huanjiang Observation and Research Station for Karst Ecosystems, Chinese Academy of Sciences, Huanjiang 547100, China; 3 University of Chinese Academy of Sciences, Beijing 100049, China; 4 Changsha Natural Resources Comprehensive Investigation Center, China Geological Survey, Changsha 410125, China; zhaoyuan1995@mail.cgs.gov.cn; 5 Huangshan Observation and Research Station for Land-Water Resources, Huangshan 245000, China; 6 Guangxi Karst Resources and Environment Research Center of Engineering Technology, Guilin 541004, China; qbohuang0108@163.com; 7 State Key Laboratory of Loess and Quaternary Geology, Institute of Earth Environment, Chinese Academy of Sciences, Xi’an 710061, China; liuji17@mails.ucas.ac.cn

**Keywords:** GRSP, carbon cycling, soil organic carbon, extracellular polymeric substance, bibliometric analysis, research hotspots

## Abstract

Glomalin-related soil protein (GRSP), a glycoprotein primarily exuded by arbuscular mycorrhizal fungi (AMF), exerts key roles in ecological processes in terrestrial ecosystems. Nevertheless, the intricate nature of GRSP, coupled with constraints in its extraction and analytical methodologies, impedes a comprehensive understanding of its compositional attributes and ecological functions. Moreover, the scope of current GRSP research has undergone significant expansion, necessitating a comprehensive synthesis in this field. Here, we employed bibliometric analysis to systematically assess research trends and hotspots in the research field of GRSP based on 840 relevant articles indexed in the Web of Science Core Collection database. Among them, key parameters evaluated encompass publications’ quantity, highly cited articles, high-frequency keywords, and historical direct citations. These analyses illuminated the state-of-the-art of GRSP research, delineated emergent trends, and provided future perspectives. Current investigations into GRSP predominantly focus on three major topics: (i) GRSP’s nature, origin, and quantification methodologies; (ii) GRSP’s key influencing factors including agricultural management practices, climate and land use change; and (iii) GRSP’s ecological functions enhancing soil aggregate stability, C sequestration, and contamination remediation. Our findings can serve as a scholarly resource for advancing inquiries into the ecological functionalities of GRSP and its prospective applications in sustainable soil management and ecological restoration.

## 1. Introduction

Glomalin-related soil protein (GRSP), a glycoprotein primarily secreted by arbuscular mycorrhizal fungi (AMF), is incorporated into fungal hyphae and spores before being released into the soil [1,2,3]. In general, GRSP encompasses three distinct fractions: total GRSP (T-GRSP), easily extractable GRSP (EE-GRSP), and difficultly extractable GRSP (DE-GRSP). T-GRSP quantifies the cumulative reservoir of GRSP within the soil, whereas EE-GRSP denotes the recently synthesized, labile fraction, and DE-GRSP represents the more recalcitrant, aged component [4,5]. Additionally, GRSP manifests several critical attributes underpinning its ecological significance: (I) a robust capacity to bind soil particulates, promoting aggregate formation and bolstering soil structural stability [6]; (II) insolubility in water and resistance to microbial decomposition, conferring prolonged persistence in soil systems [7]; (III) the capability to chelate heavy metals via diverse chemical functional groups, thereby contributing to the amelioration of soil contamination [8]; and (IV) the augmentation of soil fertility and stimulation of plant growth, highlighting its role in sustaining soil health [9]. Recently, investigations into GRSP have markedly broadened in scope, underscoring the imperative for a thorough consolidation and synthesis of advancements within this field [10,11,12].

Bibliometric analysis integrates statistical methodologies and computational techniques to analyze extensive bibliographic data encompassing keywords, citations, and references, which are interconnected and exhibit similarities across distinct information entities [13,14]. Through advanced visualization techniques, such as network diagrams and conceptual frameworks, this approach methodically elucidates the interconnections and structural organization of these entities. It serves as a powerful tool for evaluating the current state of research, pinpointing key research hotspots, and discerning emerging trends within a specified discipline or field [13,14].

To the best of our knowledge, this is the first bibliometric study to provide a comprehensive analysis of both the current state and historical development trends of GRSP research. Employing the Web of Science Core Collection database, we analyzed 840 relevant publications from 1996 to 2022 with the “*bibliometrix*” package (version 4.3.0) in R (version 4.4.1). The analysis was conducted from four key aspects: publication growth, highly cited articles, high-frequency keywords, and historical direct citations. Based on the bibliometric results, we systematically reviewed and summarized the research status, emergent hotspots, historical development trends, and future perspectives in the research field of GRSP. This study aims to establish a foundational basis for future studies exploring the nexus between GRSP dynamics and soil carbon (C) sequestration, nutrient cycling, soil health and productivity, and broader ecosystem services.

## 2. Materials and Methods

### 2.1. Data Sources and Search Strategy

In this study, scientific publications were procured from the Web of Science Core Collection database, with the most recent access dated 19 February 2023. The Web of Science platform is renowned for its comprehensive and reliable access to high-impact scientific literature, supported by robust analytical capabilities [13]. The Web of Science Core Collection was queried to retrieve studies pertinent to GRSP using the search string: “TS = (soil AND (glomalin-related soil protein OR GRSP))”. The temporal scope spanned 26 years (1996–2022), encompassing exclusively articles published in English (Figure S1). Nevertheless, this database still exhibits inherent limitations. Publications by researchers in sub-disciplinary journals insufficiently represented in widely utilized tools may not be comprehensively tracked. We recognize that the database employed may suffer from restricted coverage, potentially missing non-indexed journals. For instance, during our literature collection process, we identified an important document absent from the database [2]. To mitigate this bias, we outline supplementary strategies implemented, such as manual searches, to enhance the completeness of our analysis.

### 2.2. Bibliometric Analysis Methods

A total of 840 relevant articles were retrieved, with metadata encompassing author names, publication year, titles, keywords, abstracts, references, and additional bibliographic information. Data compilation, mapping, and visualization were performed using R (version 4.4.1) in conjunction with the “*bibliometrix*” (version 4.3.0) and “*wordcloud2*” (version 0.2.1) packages [13]. The bibliometric analysis was designed to elucidate the research status, pinpoint critical hotspots, and trace historical trajectories of GRSP through four primary aspects including publication growth, highly cited articles, high-frequency keywords, and historical direct citations.

First, we quantified the number of publications on GRSP using the global database and mapped the growth trends of publications over the past 26 years (1996–2022). This analysis can reflect the development status and tendency of the research field over time.

Second, we identified and summarized the top 10 highly cited articles within the local dataset (literature related to GRSP from 1996 to 2022). Local Citations (LC) served as the metric for ranking articles by their relative significance and pertinence, with elevated LC values denoting heightened relevance within the dataset.

Third, we analyzed high-frequency keywords, presenting them through several visualizations including word clouds, co-occurrence networks, clustering maps, and conceptual frameworks. In the word cloud, font size correlates with keyword frequency, while in co-occurrence networks, circle diameter reflects the degree of centrality of keywords [15]. Degree centrality, which measures the centrality of the nodes within a network, serves as an indicator of a keyword’s importance, with greater centrality indicating enhanced significance [13]. Networks and visualizations were color-coded to represent different clusters, and the clustering and conceptual frameworks were derived from keyword co-occurrence networks using multiple correspondence analysis. Proximal positioning of keywords on these maps signifies stronger interrelations, with the thickness of connecting lines denoting the intensity of co-occurrence [16].

Finally, a historical direct citation map was constructed to discern the most impactful and highly cited articles shaping the field’s development [13,17].

## 3. Results and Discussion

### 3.1. Tendency and Growth of Publications in GRSP Research

**Figure 1:**
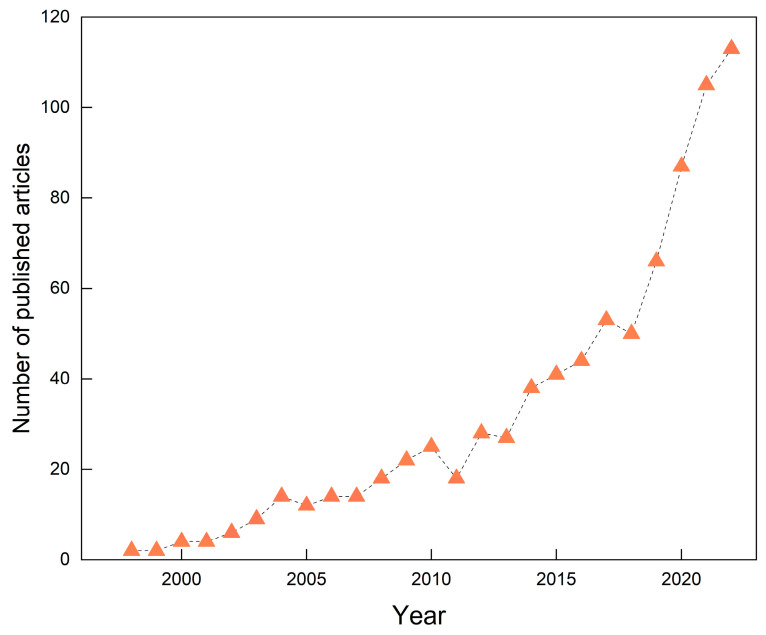
Global trends in the number of published articles in the field of glomalin-related soil protein (GRSP) from 1996 to 2022.

A consistent annual rise in the number of publications typically reflects growing attention and robust research activity within the field, facilitating the anticipation of prospective research trajectories. Figure 1 illustrates the annual number of publications in GRSP research from 1996 to 2022. Over this period, a total of 840 relevant articles were published, exhibiting a pronounced upward publication tendency and growth over time. The number of publications reached its peak in 2022, with 113 articles, accounting for 13.5% of the total corpus. Notably, the most significant growth occurred within the last five years (2018–2022), indicating that the field of GRSP research is gaining increasing attention and is currently experiencing a period of rapid development.

### 3.2. Top 10 Highly Cited Articles on GRSP

An analysis of the top 10 highly cited articles on GRSP (1996–2022) from the local dataset (Table 1 and Table 2) delineated three principal research domains closely linked to GRSP:

**(1) Contributions of GRSP to aggregate formation and C and nutrient pools in soils.** Wright and Upadhyaya [3] pioneered the demonstration of a positive correlation between GRSP concentration and aggregate stability across diverse soil types. GRSP, often referred to as “super glue”, can bind soil particles, facilitating the formation of macroaggregates, which play crucial roles in C sequestration [18]. Within 1–2 mm aggregates, GRSP’s direct stabilizing effect exceeded that of AMF hyphae, underscoring its pivotal role in hyphal-mediated aggregation stabilization mechanisms [6]. Subsequently, Rillig and Mummey [19] proposed a hierarchical model of mycorrhizal contributions to soil aggregation, emphasizing the roles of AMF hyphae and GRSP. Moreover, the hyphal association enhanced GRSP’s resistance to microbial decomposition, thereby increasing its stability in soils [3]. Moreover, GRSP constituted approximately 4–5% of the total soil C and nitrogen (N), with turnover times spanning 6 to 42 years, significantly bolstering soil nutrient reservoirs [7]. Under nutrient-limited conditions, GRSP could serve as a source of C and N for microbial decomposition and mineralization, supplying C and N [20]. Interestingly, Driver et al. [1] elucidated that GRSP is predominantly released into the soil via hyphae rather than being directly secreted by AMF. These studies verified the important contributions of GRSP to soil aggregate formation, stabilization, and C and N retention;

**(2) Factors influencing GRSP accumulation in soils.** GRSP accumulation hinges on the balance between its production and decomposition, which is modulated by an array of biotic and abiotic factors, including AMF abundance and community composition [21,22], plant traits [23], soil physicochemical properties [24], and land use changes [20]. For instance, host plants indirectly influenced GRSP production by altering the allocation of photosynthetic products to AMF [25]. Soil properties, such as clay content, can affect GRSP decomposition by providing physical protection, attenuating GRSP decomposition, and influencing its persistence [26]. Land use changes can affect the hyphal network and AMF abundance and then drive GRSP dynamics [25]. In addition, Rillig et al. [20] documented significant variations in GRSP content across cropland, forest, and plantation ecosystems, with forests exhibiting the highest GRSP concentrations and croplands the lowest;

**(3) Role of GRSP in remediating heavy metal-contaminated soils.** González-Chávez et al. [27] demonstrated GRSP’s capacity to chelate toxic elements such as copper (Cu) through reversible reactions. In addition, Khan [28] further underscored GRSP’s efficacy in adsorbing and immobilizing heavy metals in contaminated soils, transforming them into non-toxic forms, thereby enhancing plant adaptability and soil health in stressful environments

Collectively, these findings suggest that the GRSP research field primarily focuses on three key directions: (i) GRSP’s role in facilitating soil aggregate formation and contributing to soil C and N pools [7]; (ii) the dynamic accumulation of GRSP, driven by both biotic and abiotic factors, such as AMF abundance and community composition [22] and land use changes [20]; and (iii) GRSP’s involvement in the ecological remediation of heavy metal-contaminated soils [28]. Notably, GRSP-derived C is more recalcitrant to microbial decomposition than labile soil organic C, thus, contributing significantly to C sequestration in terrestrial ecosystems.

**Table 1:** Top 10 most highly cited papers on glomalin-related soil protein (GRSP) in the global database from 1996 to 2022.

Rank	First Author	Year	Journal	Title	Total Citations
1	Rillig M.C. [19]	2006	*New Phytol*	Mycorrhizas and soil structure	911
2	Wright S.F. [3]	1998	*Plant Soil*	A survey of soils for aggregate stability and glomalin, a glycoprotein produced by hyphae of arbuscular mycorrhizal fungi	772
3	Rillig M.C. [21]	2004	*Can J Soil Sci*	Arbuscular mycorrhizae, glomalin, and soil aggregation	551
4	Wilson G.W.T. [22]	2009	*Ecol Lett*	Soil aggregation and carbon sequestration are tightly correlated with the abundance of arbuscular mycorrhizal fungi: results from long-term field experiments	470
5	Khan A.G. [28]	2005	*J Trace Elem Med Bio*	Role of soil microbes in the rhizospheres of plants growing on trace metal contaminated soils in phytoremediation	383
6	Rillig M.C. [7]	2001	*Plant Soil*	Large contribution of arbuscular mycorrhizal fungi to soil carbon pools in tropical forest soils	381
7	Rillig M.C. [29]	2004	*Ecol Lett*	Arbuscular mycorrhizae and terrestrial ecosystem processes	380
8	Rillig M.C. [6]	2002	*Plant Soil*	The role of arbuscular mycorrhizal fungi and glomalin in soil aggregation: Comparing effects of five plant species	341
9	González-Chávez M.C. [27]	2004	*Environ Pollut*	The role of glomalin, a protein produced by arbuscular mycorrhizal fungi, in sequestering potentially toxic elements	311
10	Driver J.D. [1]	2005	*Soil Biol Biochem*	Characterization of glomalin as a hyphal wall component of arbuscular mycorrhizal fungi	239

**Table 2:** Top 10 most highly cited papers on glomalin-related soil protein (GRSP) in the local database from 1996 to 2022.

Rank	First Author	Year	Journal	Title	Local Citations
1	Wright S.F. [3]	1998	*Plant Soil*	A survey of soils for aggregate stability and glomalin, a glycoprotein produced by hyphae of arbuscular mycorrhizal fungi	452
2	Rillig M.C. [21]	2004	*Can J Soil Sci*	Arbuscular mycorrhizae, glomalin, and soil aggregation	318
3	Rillig M.C. [7]	2001	*Plant Soil*	Large contribution of arbuscular mycorrhizal fungi to soil carbon pools in tropical forest soils	223
4	Driver J.D. [1]	2005	*Soil Biol Biochem*	Characterization of glomalin as a hyphal wall component of arbuscular mycorrhizal fungi	181
5	Lovelock C.E. [24]	2004	*J Ecol*	Soil stocks of glomalin produced by arbuscular mycorrhizal fungi across a tropical rain forest landscape	145
6	Rillig M.C. [6]	2002	*Plant Soil*	The role of arbuscular mycorrhizal fungi and glomalin in soil aggregation: Comparing effects of five plant species	141
7	Rillig M.C. [20]	2003	*Plant Soil*	Glomalin, an arbuscular–mycorrhizal fungal soil protein, responds to land–use change	140
8	Rillig M.C. [19]	2006	*New Phytol*	Mycorrhizas and soil structure	136
9	González-Chávez M.C. [27]	2004	*Environ Pollut*	The role of glomalin, a protein produced by arbuscular mycorrhizal fungi, in sequestering potentially toxic elements	125
10	Treseder K.K. [25]	2007	*Soil Sci Soc Am J*	Glomalin in ecosystems	120

### 3.3. High-Frequency Keywords About GRSP

**Figure 2:**
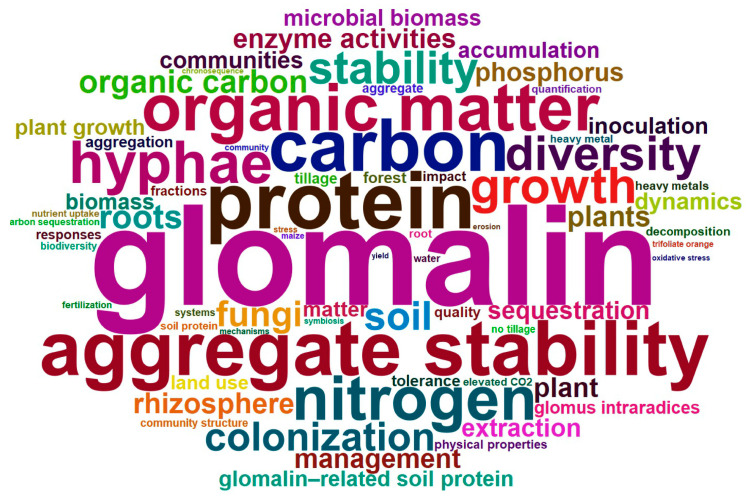
High-frequency keywords cloud map of glomalin-related soil protein (GRSP) from 1996 to 2022.

**Figure 3:**
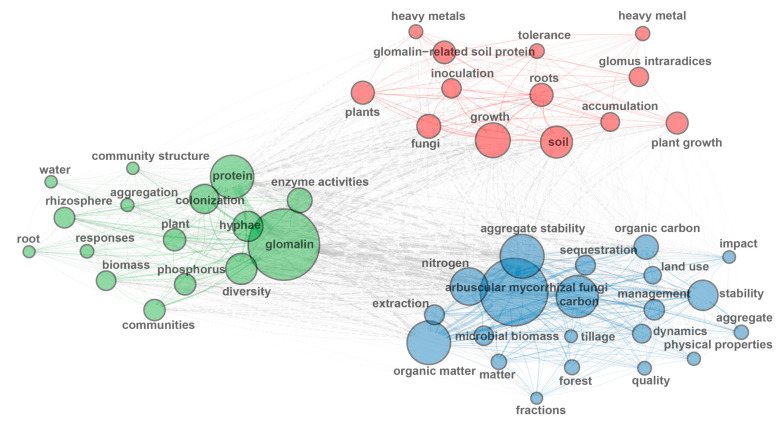
High-frequency keywords co-occurrence network about glomalin-related soil protein (GRSP) from 1996 to 2022.

**Figure 4:**
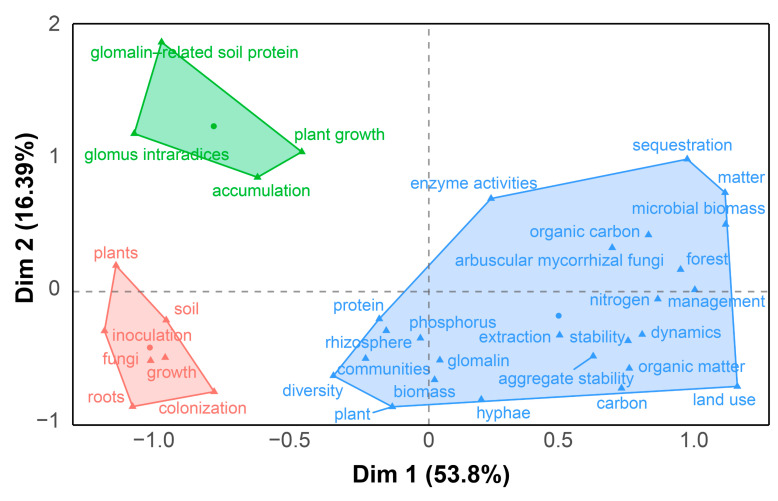
Conceptual map and high-frequency keywords clusters about glomalin-related soil protein (GRSP) from 1996 to 2022. Different colors represent distinct themes, with keywords grouped under the same color indicating similar topics; Circles represent cluster centers; Triangles represent individual high-frequency keywords.

Analysis of the top 100 high-frequency keywords from 840 GRSP articles (Figure 2, Figure 3 and Figure 4; Table 3) provides a comprehensive overview of research hotspots in the field of GRSP research. The keyword word cloud visually delineates the importance of keywords through variations in font size, whereby larger fonts denote higher keyword frequencies, typically reflecting greater topical prominence within the research field. Co-occurrence analysis extends beyond individual keyword frequency by exploring interrelationships between keyword pairs. Frequent co-occurrence of two keywords indicates a robust thematic linkage, enabling researchers to elucidate the conceptual framework of the research domain, identify major thematic clusters, and map interconnected pathways among research topics [13,14]. The keyword co-occurrence network (Figure 2 and Figure 3; Table 3) identified the top 10 high-frequency keywords, including *glomalin*, *arbuscular mycorrhizal fungi*, *protein*, *aggregate stability*, *carbon*, *organic matter*, *nitrogen*, *hyphae*, *growth*, and *diversity*. These terms suggest that GRSP research primarily focused on the origins, relationships with aggregate stability, and contributions to soil C and N accrual. Moreover, Figure 2 and Figure 3 further revealed current GRSP research as an interdisciplinary nexus spanning pedology, microbiology, ecology, and environmental science.

Keyword clustering elucidates the interrelationships and hierarchical structure among distinct research themes. These clustering results can be used to construct a knowledge map of the research field, allowing researchers to visualize thematic linkages and shed insight into the field’s comprehensive intellectual framework [13,14]. The keyword clustering and conceptual structure maps (Figure 4) categorized these high-frequency keywords into three distinct clusters. Dimension 1 (Dim 1) and Dimension 2 (Dim 2), respectively, accounted for 53.8% and 16.39% of the total variance, yielding a cumulative explained variance (70.19%). A detailed analysis of these three clusters aligned seamlessly with findings from Figure 2 and Figure 3:

**(1) Cluster 1 focused on the nature and source of GRSP.** This cluster featured high-frequency keywords such as *protein*, *rhizosphere*, *hyphae*, *community structure*, *diversity*, *root*, and *plant* (Figure 4), emphasizing that GRSP’s nature is proteins secreted by AMF hyphae associated with host plant root, correlating with microbial community structure and diversity in the rhizosphere [1,21,22];

**(2) Cluster 2 focused on GRSP’s influence on soil aggregate stability, C and N dynamics, and responses to environmental changes.** The cluster featured high-frequency keywords such as *aggregate stability*, *organic carbon*, *nitrogen*, *land use*, *management*, *elevated CO_2_*, and *microbial biomass* (Figure 4), highlighting the close relation between GRSP and aggregate stability, and pivotal contributions to C and N pools, which can be significantly influenced by agricultural management practices, land use, and climate change [7,19,20,30].

**(3) Cluster 3 focused on the remediation potential of GRSP in heavy metal-contaminated soils.** This cluster featured high-frequency keywords such as *heavy metals*, *plant growth*, *tolerance*, *inoculation*, *accumulation*, and *growth* (Figure 4), reflecting its capacity to mitigate toxicity and bolster plant performance in stress environments [27,28].

Together, high-frequency keywords analysis delineated three core research themes in the research field of GRSP: (i) the nature and source of GRSP; (ii) the roles of GRSP in soil aggregate stability and C and N dynamics, and their influencing factors; and (iii) the capacity of GRSP in remediating heavy metal-contaminated soils.

### 3.4. Historical Direct Citations About GRSP

**Figure 5:**
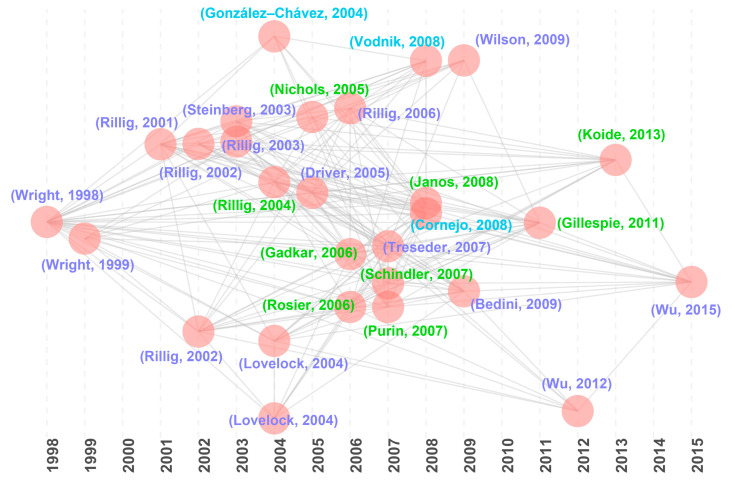
Historical direct citations map about glomalin-related soil protein (GRSP) from 1996 to 2022 [1,3,4,5,6,7,19,20,21,22,23,24,25,26,27,30,31,32,33,34,35,36,37,38,39,40,41,42].

Historical direct citation analysis identified 28 milestone articles (Figure 5; Table 4) that have profoundly shaped GRSP research, aligning closely with the highly cited articles (Table 1 and Table 2). These influential studies cluster into three key directions:

**(1) Discovery, origins, and characteristics of GRSP**

Wright and Upadhyaya [2] for the first time identified glomalin as a glycoprotein secreted by AMF hyphae. Steinberg and Rillig [32] explored the decomposition of AMF hyphae and GRSP, noting a 60% reduction in hyphal length versus a 25% decline in GRSP content after 150 d incubation. Although this study might overestimate decomposition rates of AMF hyphae and GRSP due to the absence of plants, it filled a gap in understanding hyphal and GRSP decomposition rates in soil. Driver et al. [1] conducted in vitro incubation of AMF and demonstrated that GRSP is predominantly associated with hyphal and spore walls, being released into the soil primarily through hyphal interactions rather than direct secretion by the AMF. Gadkar and Rillig [34] employed liquid chromatography-mass spectrometry (LC-MS/MS) to sequence GRSP bands reactive to the specific antibody MAb32B11, identifying homology with Hsp60, elucidating its thermal stability and iron-binding capacity.

**(2) Methodological advances in GRSP quantification**

Rillig [21] introduced the term “glomalin-related soil protein (GRSP)” to describe all proteins extracted, challenging the prior assumption that glomalin was just a specific protein or protein group. Traditional GRSP measurement methods, such as the Bradford method, are based on the premise that most non-thermostable soil proteins are destroyed during the rigorous extraction process. However, this assumption has yet to be thoroughly validated. Rosier et al. [33] tested the impact of adding known amounts of glycoproteins (e.g., bovine serum albumin) or specific litter sources to soil samples, and discovered the limitations of the Bradford method, as non-glomalin proteins persist post extraction. This raises concerns about the accuracy of GRSP detection and quantification, particularly in soils with high organic matter content. Schindler et al. [36] further demonstrated that GRSP extracts prepared with current methods contain a mixture of proteins along with a significant amount of excess humic acid. Subsequently, Janos et al. [39] extended this work to other soils and modified the widely used sodium citrate extraction protocol, recommending three key adjustments: (i) using equal volumes of extraction solution, (ii) extracting under the same autoclaving time and removing samples promptly at the cycle end, and (iii) immediately centrifuging after autoclaving to separate the supernatant from the soil.

In addition, Gillespie et al. [41] utilized synchrotron-based X-ray Absorption Near Edge Structure (XANES) and Pyrolysis-Field Ionization Mass Spectrometry (Py-FIMS) to comprehensively analyze the structure of GRSP. The results showed that GRSP contains both proteins and numerous impurities such as various hydrophobic compounds (e.g., fatty acids and lignin), suggesting that the current GRSP extraction methods (Bradford method) yield overestimation risks. Additionally, Enzyme-Linked ImmunoSorbent Assay (ELISA) is another alternative method for detecting GRSP, employing the MAb32B11 antibody [2]. However, the binding specificity of the MAb32B11 antibody remains unclear, and it is speculated to interact with multiple proteins present in *Glomus intraradices* spores [41].

**(3) Roles of GRSP in aggregate stability, C sequestration, and heavy metal immobilization**

**(i) Aggregate stability:** Wright and Upadhyaya [3] observed a significant positive correlation between GRSP and aggregate stability. Rillig et al. [6] further investigated the effects of AMF and other biotic factors on soil aggregates, contrasting the relative importance of GRSP with other factors such as AMF hyphae, root length, and vegetation cover. They found that GRSP, root length, and vegetation cover all significantly influence the formation of water-stable aggregates, with GRSP exerting a more direct effect than AMF hyphae. Moreover, GRSP plays a critical role in a hyphae-mediated mechanism of soil aggregate stabilization in 1–2 mm aggregates, surpassing hyphal contributions [21]. As a long-term binder, GRSP helps cement soil particles, thus, enhancing aggregate stability [40]. Yang et al. found that long-term fertilization promotes the accumulation of GRSP by altering its chemical composition (aromatic C) mediated by AMF while concurrently strengthening the protection of macroaggregates, as evidenced by a 29-year fertilization experiment [11]. This investigation provides a feasible approach to improve soil quality and C sequestration in sustainable agriculture development.

**(ii) C sequestration:** Rillig et al. [7] reported GRSP’s 4–5% contribution to soil C pools in tropical forests with turnover times of years to decades, highlighting its pivotal role in long-term C sequestration. In a follow-up study, Rillig et al. [20] illustrated higher GRSP storage in forests compared to croplands, contributing 3.77–7.84% to soil organic C. Next, Lovelock et al. [24] quantified GRSP accumulation in a lowland tropical rainforest along a nutrient gradient and revealed the average GRSP content in the topsoil was 3.94 ± 0.16 mg cm^−3^ (1.45 Mg C ha^−1^), accounting for 3.2% of the total C in the 0–10 cm soil layer, with higher EE–GRSP concentrations in soils rich in calcium, phosphorus, and potassium. In the early stages of secondary succession, GRSP can directly contribute to organic C to stabilize the soil C pool, thereby deepening our understanding of its ecological roles in C sequestration and soil structure improvement [43]. Additionally, Wu et al. [42] illustrated a significant positive correlation between GRSP with *β*-glucosidase in the citrus rhizosphere, suggesting that GRSP may play a role in the release of glucose, thereby supporting metabolically active microbial biomass in soils. This emphasizes the importance of considering *β*-glucosidase as a factor influencing GRSP accumulation [44].

**(iii) Heavy metal immobilization:** González-Chávez et al. [27] explored the interactions between potentially toxic elements and GRSP in two contaminated soils and found that GRSP played a role in the immobilization of various heavy metals via reversible reactions. Cornejo et al. [38] confirmed the capacity of GRSP to immobilize Cu and zinc (Zn), with GRSP-bound Cu ranging from 3.76 to 89.0 mg g^−1^, accounting for 1.44–27.5% of total soil Cu. Moreover, Vodnik et al. [37] reported that GRSP-bound lead (Pb) accounted for 0.8–15.5% of total Pb, preferentially binding Pb over Zn. Wang et al. [45] through subsequent investigations, determined that a composite index of nine heavy metals exhibited a significant positive correlation with GRSP content, indicating that GRSP may serve as a bioindicator of pollution levels in mangrove wetlands. The widespread distribution of GRSP in sediments and suspended particles amplifies the immobilization capacity for heavy metals within the aquatic ecosystem of mangrove forests. Therefore, GRSP demonstrates excellent buffering potential against acute heavy metal pollution emergencies, which will help us understand its ecological regulatory role in the remediation of heavy metal-contaminated soils [46,47].

Collectively, these studies underscored GRSP’s multifaceted ecological significance and the need for improving extraction and quantification methods, advancing knowledge of the underlying mechanisms, and investigating the broader ecological functions and applications of GRSP.

## 4. Conclusions and Perspectives

This investigation delineated three predominant directions in the current GRSP research field: (i) GRSP’s nature, origin, and quantification methodologies; (ii) GRSP’s key influencing factors including agricultural management practices and climate and land use change; and (iii) GRSP’s ecological functions enhancing soil aggregate stability, C sequestration, and contamination remediation.

To advance comprehension of GRSP’s nature, ecological functions, and mechanisms, future research could prioritize the following perspectives:

**(1) Refinement of extraction and quantification methods**

Currently, the Bradford method remains a prevalent method for GRSP quantification [2]. However, the impurity of the extracted glycoproteins, which results from the co-extraction of proteins and non-protein compounds from diverse origins, introduces significant challenges. Numerous studies have examined potential interference sources and proposed refined methodologies to address these issues [48,49]. Despite such progress, the inherent complexity of GRSP continues to impede precise quantification, underscoring the necessity for more sophisticated analytical techniques. Notably, advanced techniques, including Scanning Electron Microscopy coupled with Energy-Dispersive X-ray spectroscopy (SEM-EDX), Fourier Transform Infrared Spectroscopy (FTIR), X-ray Photoelectron Spectroscopy (XPS), X-ray Diffraction (XRD), DNA Stable-Isotope Probing (DNA-SIP), and Nano-scale Secondary Ion Mass Spectrometry (NanoSIMS), have been employed to analyze and identify the main components, structure, and stability of GRSP [8,50,51,52,53,54], thereby advancing our understanding. Future efforts should optimize extraction protocols and enhance the accuracy of quantification, focusing on clarifying GRSP-AMF interrelationships, delineating distinct GRSP’s roles, and leveraging metabolomics to elucidate GRSP’s origin, structure, and composition [44,54].

**(2) Elucidation of mechanisms and dynamics**

A deeper understanding of GRSP’s involvement in ecological processes demands a shift from correlative analyses to mechanistic and causal frameworks. Such insights will bolster the strategic deployment of GRSP’s ecological functions such as soil C sequestration in terrestrial ecosystems [54,55,56,57].

**(3) Soil remediation and pollution abatement**

Beyond its established role of GRSP in heavy metal remediation, further exploration is needed into GRSP’s efficacy against organic pollutants, petroleum hydrocarbons, and saline–alkaline conditions. Investigations could extend to pesticide- and radionuclide-contaminated soils, emphasizing mechanistic underpinnings [58,59].

**(4) Contributions to soil N reservoirs**

While GRSP’s role in soil C pools is well-documented, its influence on N dynamics remains underexplored. GRSP contains N, which may contribute a lot to the soil N pool. Additionally, GRSP can be decomposed and mineralized by microorganisms in N-limited soils, thus, serving as a N source. Future studies may quantify GRSP’s contributions to soil N pools and examine its interactions with soil nutrients, microbial communities, enzymatic activities, and plant productivity [55,57].

Addressing these research gaps will deepen our understanding of GRSP’s ecological significance and mechanisms, facilitating its optimized application in environmental stewardship and sustainable ecosystems.

## Figures and Tables

**Table 3:** Keywords co-occurrence network analysis about glomalin-related soil protein (GRSP) from 1996 to 2022.

Rank	Keywords	Occurrences	Degree Centrality
1	*glomalin*	311	1.000
2	*arbuscular mycorrhizal fungi*	259	0.845
3	*protein*	179	0.562
4	*aggregate stability*	171	0.553
5	*carbon*	162	0.530
6	*organic matter*	148	0.501
7	*nitrogen*	137	0.456
8	*hyphae*	131	0.401
9	*growth*	112	0.360
10	*diversity*	107	0.344

**Table 4:** Historical direct citations about glomalin-related soil protein (GRSP) from 1996 to 2022.

Rank	First Author	Year	Title	Local Total Citations
1	Wright S.F. [3]	1998	A survey of soils for aggregate stability and glomalin, a glycoprotein produced by hyphae of arbuscular mycorrhizal fungi	452
2	Wright S.F. [31]	1999	Changes in aggregate stability and concentration of glomalin during tillage management transition	85
3	Rillig M.C. [7]	2001	Large contribution of arbuscular mycorrhizal fungi to soil carbon pools in tropical forest soils	223
4	Rillig M.C. [23]	2002	Glomalin production by an arbuscular mycorrhizal fungus: A mechanism of habitat modification?	84
5	Rillig M.C. [6]	2002	The role of arbuscular mycorrhizal fungi and glomalin in soil aggregation: Comparing effects of five plant species	141
6	Rillig M.C. [20]	2003	Glomalin, an arbuscular-mycorrhizal fungal soil protein, responds to land-use change	140
7	Steinberg P.D. [32]	2003	Differential decomposition of arbuscular mycorrhizal fungal hyphae and glomalin	86
8	Rillig M.C. [21]	2004	Arbuscular mycorrhizae, glomalin, and soil aggregation	318
9	Lovelock C.E. [24]	2004	Soil stocks of glomalin produced by arbuscular mycorrhizal fungi across a tropical rain forest landscape	145
10	González-Chávez M.C. [27]	2004	The role of glomalin, a protein produced by arbuscular mycorrhizal fungi, in sequestering potentially toxic elements	125
11	Lovelock C.E. [30]	2004	Using glomalin as an indicator for arbuscular mycorrhizal hyphal growth: An example from a tropical rain forest soil	63
12	Driver J.D. [1]	2005	Characterization of glomalin as a hyphal wall component of arbuscular mycorrhizal fungi	181
13	Nichols K.A. [26]	2005	Comparison of glomalin and humic acid in eight native U.S. soils	57
14	Rosier C.L. [33]	2006	Glomalin-related soil protein: Assessment of current detection and quantification tools	95
15	Gadkar V. [34]	2006	The arbuscular mycorrhizal fungal protein glomalin is a putative homolog of heat shock protein 60	86
16	Rillig M.C. [19]	2006	Mycorrhizas and soil structure	136
17	Purin S. [35]	2007	The arbuscular mycorrhizal fungal protein glomalin: Limitations, progress, and a new hypothesis for its function	64
18	Schindler F.V. [36]	2007	Chemical characteristics of glomalin-related soil protein (GRSP) extracted from soils of varying organic matter content	111
19	Treseder K.K. [25]	2007	Glomalin in Ecosystems	120
20	Vodnik D. [37]	2008	The contribution of glomalin-related soil protein to Pb and Zn sequestration in polluted soil	81
21	Cornejo P. [38]	2008	Glomalin-related soil protein in a Mediterranean ecosystem affected by a copper smelter and its contribution to Cu and Zn sequestration	100
22	Janos D.P. [39]	2008	Glomalin extraction and measurement	61
23	Bedini S. [40]	2009	Changes in soil aggregation and glomalin-related soil protein content as affected by the arbuscular mycorrhizal fungal species *Glomus mosseae* and *Glomus intraradices*	105
24	Wilson G.W.T. [22]	2009	Soil aggregation and carbon sequestration are tightly correlated with the abundance of arbuscular mycorrhizal fungi: Results from long-term field experiments	74
25	Gillespie A.W. [41]	2011	Glomalin-related soil protein contains non-mycorrhizal-related heat-stable proteins, lipids and humic materials	95
26	Wu Q.S. [42]	2012	Spatial distribution of glomalin-related soil protein and its relationships with root mycorrhization, soil aggregates, carbohydrates, activity of protease and *β*-glucosidase in the rhizosphere of *Citrus unshiu*	57
27	Koide R.T. [4]	2013	Behavior of Bradford-reactive substances is consistent with predictions for glomalin	57
28	Wu Q.S. [5]	2015	Arbuscular mycorrhiza mediates glomalin-related soil protein production and soil enzyme activities in the rhizosphere of trifoliate orange grown under different P levels	57

## Data Availability

The data used to support the findings of this study can be made available by the corresponding authors upon request.

## Supplementary Materials

The following supporting information can be downloaded at: www.mdpi.com/article/10.3390/microorganisms13040740/s1, Figure S1: The study selection and flow chart of the research framework.
