# Supplementary figures and images for: Advances, Challenges, and Perspectives in Glomalin-Related Soil Protein Research
Source: Microorganisms. 2025 Mar 25;13(4):740. doi: 10.3390/microorganisms13040740 (PMC12029919; doi:10.3390/microorganisms13040740)

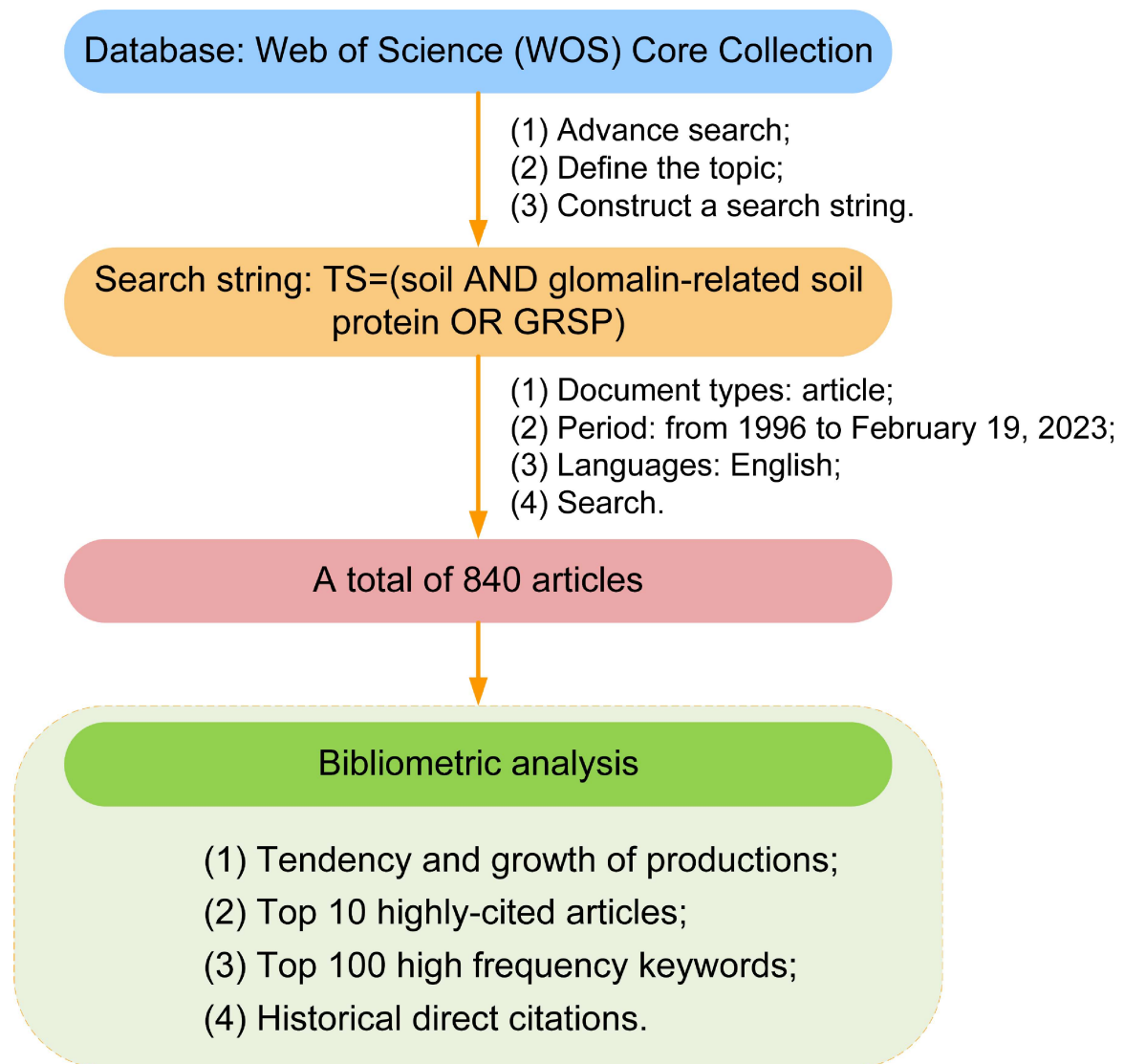

Fig. S1 The study selection and flow chart of the research framework.

Supplement: Supplementary file 1 [file microorganisms-13-00740-s001.zip › microorganisms-3521155-supplementary.pdf]
